# Promoter insertion leads to polyembryony in mango — a case of convergent evolution with citrus

**DOI:** 10.1093/hr/uhad227

**Published:** 2023-11-08

**Authors:** Chandra Bhan Yadav, Ada Rozen, Ravit Eshed, Mazal Ish-Shalom, Adi Faigenboim, Natalie Dillon, Ian Bally, Matthew Webb, David Kuhn, Ron Ophir, Yuval Cohen, Amir Sherman

**Affiliations:** Institute of Plant Sciences, Volcani Research Center, Derech Hamacabim 68, Rishon LeZion, P.O. Box 15159, 7528809, Israel; Department of Genetics, Genomics, and Breeding, NIAB-EMR, East Malling, ME19 6BJ, United Kingdom; Institute of Plant Sciences, Volcani Research Center, Derech Hamacabim 68, Rishon LeZion, P.O. Box 15159, 7528809, Israel; Institute of Plant Sciences, Volcani Research Center, Derech Hamacabim 68, Rishon LeZion, P.O. Box 15159, 7528809, Israel; Institute of Plant Sciences, Volcani Research Center, Derech Hamacabim 68, Rishon LeZion, P.O. Box 15159, 7528809, Israel; Institute of Plant Sciences, Volcani Research Center, Derech Hamacabim 68, Rishon LeZion, P.O. Box 15159, 7528809, Israel; Department of Agriculture and Fisheries, Horticulture and Forestry Science, 28 Peters St, Mareeba, QLD 4880, Australia; Department of Agriculture and Fisheries, Horticulture and Forestry Science, 28 Peters St, Mareeba, QLD 4880, Australia; Department of Agriculture and Fisheries, Horticulture and Forestry Science, Ecosciences Precinct, 41 Boggo Road, Dutton Park, Brisbane, QLD 4001, Australia; Subtropical Horticulture Research Station, USDA-ARS, 13601 Old Cutler Rd, Coral Gables, FL 33158, United States; Institute of Plant Sciences, Volcani Research Center, Derech Hamacabim 68, Rishon LeZion, P.O. Box 15159, 7528809, Israel; Institute of Plant Sciences, Volcani Research Center, Derech Hamacabim 68, Rishon LeZion, P.O. Box 15159, 7528809, Israel; Institute of Plant Sciences, Volcani Research Center, Derech Hamacabim 68, Rishon LeZion, P.O. Box 15159, 7528809, Israel

## Abstract

Sexual reproduction in plants is the main pathway for creating new genetic combinations in modern agriculture. In heterozygous plants, after the identification of a plant with desired traits, vegetative propagation (cloning) is the primary path to create genetically uniform plants. Another natural plant mechanism that creates genetically uniform plants (clones) is apomixis. In fruit crops like citrus and mango, sporophytic apomixis results in polyembryony, where seeds contain multiple embryos, one of which is sexually originated and the others are vegetative clones of the parent mother tree. Utilizing the mango genome and genetic analysis of a diverse germplasm collection, we identified *MiRWP* as the gene that causes polyembryony in mango. There is a strong correlation between a specific insertion in the gene’s promoter region and altered expression in flowers and developing fruitlets, inducing multiple embryos. The *MiRWP* gene is an ortholog of *CitRWP* that causes polyembryony in citrus. Based on the data, we speculate that promoter insertion events, which occurred independently in citrus and mango, induced nucellar embryogenesis. The results suggest convergent evolution of polyembryony in the two species. Further work is required to demonstrate the utility of these genes (mango and citrus) in other biological systems as a tool for the clonal production of other crops.

## Introduction

Sexual reproduction is the main pathway for the creation of genetic variations. Plant meiosis generates new combinations of genetic material through recombination and chromosome segregation [1], creating progenies with different phenotypes. In contrast, in modern agriculture, one of the important goals is uniform plant material that responds to growth conditions. Specific genotypes with improved horticultural properties, selected from the hybrid progeny of a breeding program, must be conserved as a “clone” and be propagated in large quantities [2]. Highly homozygous vegetable cultivars are often genetically stabilized through backcrossing schemes to enable them to “breed true” to the mother plant from seeds, creating genetically uniform plants. However, cultivars in other plants, such as fruit trees, generated by sexual reproduction are often heterozygous and do not breed true. To preserve and multiply selected individuals in such heterozygous plant systems, vegetative (asexual) reproduction is needed to propagate genetically identical individuals (clones). Most agricultural practices for creating clones in fruit trees, some forest trees, and numerous other species are based on vegetative propagation [3, 4] through cuttings, rooting, grafting, and tissue culture. However, there are many crops where vegetative reproduction is difficult [2, 5] and some in which it is practically impossible [6].

Another asexual reproductive strategy that is naturally found in some angiosperm is apomixis. Apomixis is defined as asexual reproduction through seeds that leads to the production of clonal progeny whose genotype is identical to that of the mother plant [7]. Apomixis mechanisms in seeds are subdivided into gametophytic or sporophytic, based on whether the embryo develops via a gametophyte (embryo sac) or directly from diploid somatic tissues (sporophytic). Apomixis is rarely obligatory as most apomictic genera have both apomictic and sexual reproduction occurring in the same plant/flower [8]. However, apomixis is rarely used for agricultural purposes [9, 10]. Manipulation of apomixis in different crops may provide an alternative way to propagate plants and may even form an alternative to hybrid seed production [11, 12]. Attempts to introgress apomixis to crops from apomictic relatives have been unsuccessful [9, 13]. Recently, a few apomictic artificial systems have been developed in crop plants [14, 15].

Apomixis occurs in at least 80 families (12%) and 300 genera (1.8%) of angiosperms [16], but besides mango and citrus, no major horticultural fruit crop species are apomictic [17]. In citrus and mango, a seed formed through apomixis may contain multiple embryos, one of which is sexually derived while the others are asexually derived and clones of the mother tree. This phenomenon called sporophytic apomixis leads to the occurrence of polyembryos and is therefore defined in citrus and mango as polyembryony (a seed with only one embryo that is a result of sexual reproduction is defined as monoembryony). In ovules of polyembryonic plants, the embryo sac develops as in sexual reproduction, and after double fertilization forms a zygotic embryo and a triploid endosperm. In parallel, diploid somatic cells (2N) begin to differentiate from the nucellar tissue to form globular adventitious nucellar embryos (or polyembryos), which can only develop into mature embryos when the complete sexual process occurs [8]. The presence of a functional endosperm is crucial, as both sexual and asexual embryos are dependent on the endosperm for nutrition.

Mango, like citrus, has both monoembryonic and polyembryonic seed types [18, 19]. Mangoes that originated from Southeast Asia are typically polyembryonic, while those from the Myanmar-Indochinese region are typically monoembryonic [20, 21]. In mango breeding programs, monoembryonic varieties are used as maternal parents to create hybrids derived from the zygotic embryo. The polyembryony trait is the basis of most mango rootstocks. Polyembryonic varieties can be propagated through seeds that produce multiple maternal clones from each seed that do not require grafting to produce genetically uniform trees. Therefore, the polyembryonic trait is used in most mango rootstocks. Mango seeds are very large, so a visual inspection can easily detect the mono/polyembryonic accessions.

The polyembryonic trait segregates in both mango and citrus, which are phylogenetically related, as a single dominant Mendelian trait [18, 19]. The citrus *CitRWP* gene was identified as causing polyembryony [19]. Enhanced expression of a *CitRWP* allele as a result of a miniature inverted-repeat transposable element (MITE) insertion leads to polyembryony [19, 22–24]. Genes from the plant-specific family of transcription factors, RWP—with the RK domain, have been shown to function in the maintenance of egg-cell identity in *Arabidopsis*. Overexpression of one of these genes promotes ectopic embryogenesis in somatic tissues [25], supporting the role of overexpression of *RWP* orthologs in citrus as the causal gene of polyembryony.

Mango developed from two centers of origin in South East Asia and North East India [20]. Mango is one of the most important fruit crops, with an annual production of more than 57 million tons and second only to banana among tropical and subtropical fruits. However, the biology of mango is understudied. The lack of genetic and genomic resources limited progress in mango research. Recently, several genomic tools for mango were created, including transcriptome data [26–28], two detailed genetic maps [29, 30], and genome sequence drafts [31–33]. Mango (*Mangifera indica*) is a true diploid by its genetics and cytogenetics. The mango genome size is ~400–440 Mbps [32, 33]. It is considered to have undergone WGD events around 70 million years ago, with specific duplicated regions and duplicated genes that were retained in the genome [32, 33].

Currently, little is known about the genes involved in mango polyembryony. In our previous efforts, we mapped the polyembryony locus to a region of mango chromosome 7 in two mapping populations [30] (chromosome number is based on the map of Luo et al. [28]). In the present study, utilizing genetic and genomic approaches, we identified the gene that causes polyembryony in mango and characterized specific monoembryonic and polyembryonic alleles.

## Results

### Histological analysis of mono- and poly-embryo development

**Figure 1:**
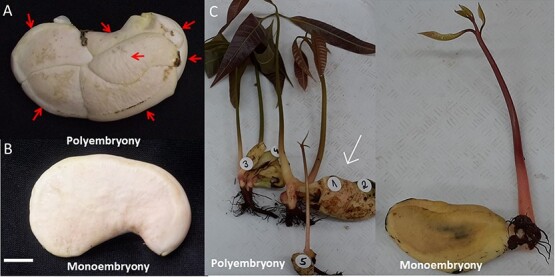
The phenotype of polyembryonic and monoembryonic seeds in mango. (**A**) Multiple embryos (each indicated by a red arrow) in a polyembryonic mango cultivar seed. The red arrows indicate the individual embryos—the scale bar is 10 mm. (**B**) A single embryo in a monoembryonic cultivar seed. (**C**) Seed germination phenotypes in polyembryonic accession (‘13-1’, Left). Multiple plantlets develop from the different embryos (the sexual one identified by PCR analysis based on specific SNP markers is marked with a white arrow). Monoembryonic accession (Shelly, Right) with a single plantlet.

After the removal of the hard endocarp and papery testa in mango seeds, the embryonic phenotype was visually determined. In polyembryonic accessions, multiple embryos appeared as a segmented mass of embryos. In monoembryonic accessions, only a single unsegmented embryo with two cotyledons filling the entire seed space was present (Fig. 1A and B). After germination, polyembryonic accessions typically have few and separate seedlings developing from a single seed (Fig. 1C), whereas in monoembryonic accessions, only a single seedling germinates from each seed. The number of polyembryonic embryos developed varies and depends on both the genetic background and environmental conditions.

**Figure 2:**
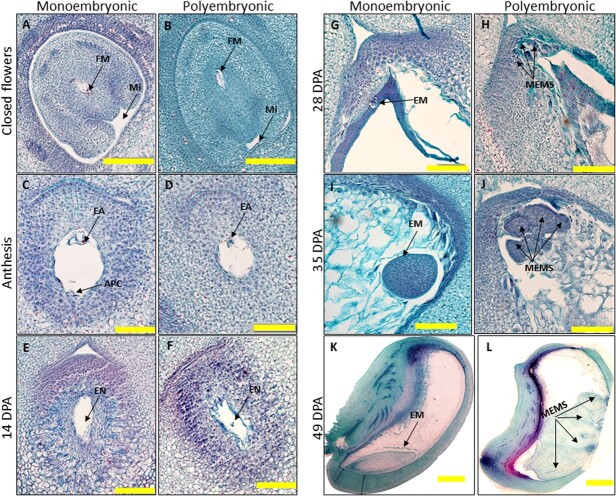
Embryo development in mono- and polyembryonic mangoes. Histological studies of the carpel and the early fruitlets at different developmental stages of embryo development. (**A**, **B**) A functional megaspore (FM) during embryo sac development embedded in nucellus at the closed flower stage; Mi—micropyle. (**C**, **D**) Sexual embryo sac showing antipodal cells (APC), egg apparatus (EA), and central cell, during anthesis. (**E**, **F**) The multinucleated syncytium region (SYN) (which was lost during tissue preparation) 14 days post-anthesis (DPA). This region develops into the endosperm; single embryo (EM) (**G**, **I**, **K**) or multiple embryos (MEMS) (**H**, **J**, **L**) development in monoembryonic (Shelly) and polyembryonic (‘Sabre’) accessions, respectively. The bar scale represents 100 μm (in **A** and **B**), 50 μm (in **C**–**J**), and 1 mm (in **K** and **L**).

To identify the differences in early embryonic development between monoembryonic and polyembryonic seeds, we studied flower and early fruit development of polyembryonic vs. monoembryonic accessions at the microscopic scale of two polyembryonic accessions (‘13-1’ and ‘Sabre’) and two monoembryonic cultivars (‘Shelly’ and ‘Omer’) (Fig. 2; Supplementary Fig. S1). Closed and open flowers showed similar early development and differentiation of the ovule and embryo sac (Fig. 2A–D). In both the monoembryonic and polyembryonic accessions, the embryo sac contains intact egg apparatus, antipodal cells, and polar nuclei. After fertilization, endosperm development was detected (Fig. 2E and F). In the polyembryonic accessions, nucellar cells situated at the micropillar region with dense cytoplasmic content and large nuclei develop into multiple nucellar embryos (Fig. 2H, J, and L), while in the monoembryonic accessions, only a single embryo developed (Fig. 2G, I, and K).

### Fine mapping of the polyembryony locus

**Figure 3:**
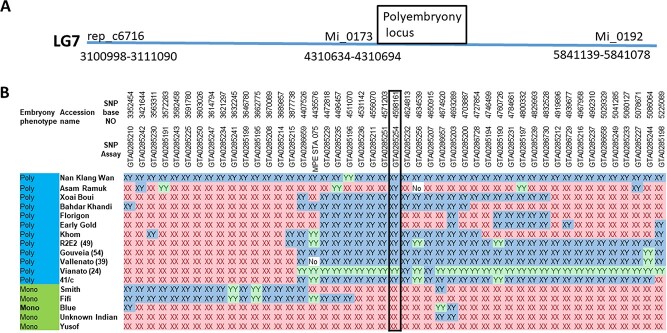
Genetic mapping of the polyembryony locus. (**A**) The genetic map of the polyembryony locus on chromosome 7 of mango is based on the information presented by Kuhn et al. [30]. (**B**) Genetic mapping of the polyembryonic locus utilizing SNP assays on germplasm collection—few accessions are presented. The location of the locus is around 44598161 bp, which is marked by the black box. Red - homozygote (XX); blue - heterozygote (XY), green - homozygote (YY). The full set of data is in Supplementary Table S4. LOD score analysis of the data is presented in Supplementary Fig. S2.

In our previous study [30], we mapped the polyembryony locus to chromosome 7 between two markers: Mango_rep_6716 positioned at 3110149–3110248 bp and Mi_0192 (position 5841139–5841078) and close to marker Mi_0173 (position 4310634–4310694 bp). These positions are based on the marker sequences identified in ‘Tommy Atkins’ genome draft [33] (Fig. 3A). As our population was relatively small with a limited number of recombinants, the polyembryony locus was mapped by combining two germplasm collections from Israel and Australia which are a mix of commercial varieties and accession that were collected from different parts of the world including India, Southeast Asia, Australia, USA, and Israel, based on the phenotype, genetic and genomic tools. A set of accessions were phenotyped for the polyembryony trait by visually examining five fruits per tree for two or more seasons. Overall, 107 monoembryonic accessions and 93 polyembryonic accessions were phenotyped (Supplementary Table S1). To narrow down the polyembryonic locus on chromosome 7 (Fig. 3A), we re-sequenced 15 mango accessions, 8 polyembryonic, and 7 monoembryonic accessions (Supplementary Table S2) by NGS Illumina technology. SNPs (Single Nucleotide Polymorphism) in the area between the flanking markers (rep_6716 and Mi_0192) that harbor the polyembryony trait (Fig. 3A) were extracted by comparison to the ‘Tommy Atkins’ (TA) reference genome [33] (Supplementary Dataset S1). Based on the assumption that polyembryonic accessions are expected to be heterozygous and monoembryonic types homozygous [18, 30], bioinformatics analysis revealed two regions with expected genotypes that correlate with the 15 accession phenotypes (Supplementary Table S1, Supplementary Dataset S1). We selected a set of SNPs that covered the two identified regions to further map the trait in the germplasm collection by high-throughput genotyping, utilizing the EP1 platform (www.fluidigm.com). In this analysis, most accessions were either from the mono or the poly haplotype, in correlation with their phenotype (Fig. 3B, Supplementary Dataset S2). However, a small subset of accessions revealed a mixed haplotype. This subset defines the area around SNP position 4598161 as the area of the polyembryony locus. This marker was the only marker with full correlation with the polyembryony trait and had the highest LOD score (Fig. 3B, Supplementary Fig. S2). We defined the locus area between 4571203 bp and 4628134 bp on chromosome 7, based on the markers surrounding marker 4598161, as the area of the polyembryony locus. This region contains six predicted genes. To identify the causative gene, markers within coding regions in all reading frames of all genes in this area were re-analyzed with the NGS data (15 sequenced genomes). Two genes, *Manin07g00350.1* and *Manin07g00,5360.1*, did not contain any SNPs in their coding region that were entirely associated with the polyembryonic trait. A first analysis using BLASTP a against plant subset database of the four other genes (*Manin07g005310.1*, *Manin07g005320.1*, *Manin07g005330.1*, *Manin07g005340.1*) showed that *Manin07g005330.1* encodes an RWP domain containing protein, similar to the *Arabidopsis* RKD family of proteins, which serve as regulators of egg-cell-related genes, and that has strong homology to the *CitRWP* gene which causes polyembryony in citrus. Based on these data and gene expression patterns (shown later), we conclude that the *Manin07g005330.1* allele is the possible cause of polyembryony in mango. We renamed the gene *MiRWP*.

### Expression analysis of the predicted gene

**Figure 4:**
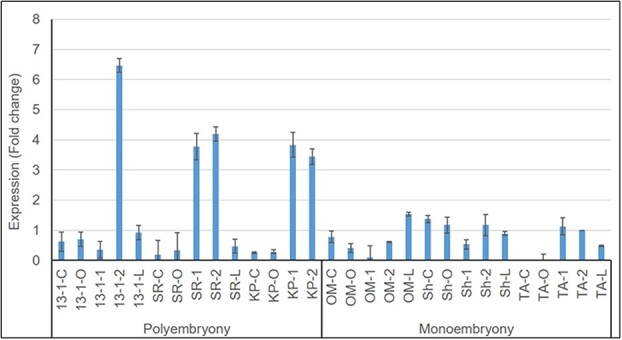
The relative expression of *MiRWP* in flowers and early fruitlet development. RNA was extracted from whole carpels (at the flowering stage) and isolated ovules (1 and 5-mm fruitlets) using qRT-PCR in polyembryonic (‘13-1’, ‘Sabre’ (SR), 'Kensington Pride' (KP)) and monoembryonic (‘Omer’ (Om), ‘Shelly’ (Sh), ‘Tommy Atkins’ (TA)) mango cultivars across various reproductive developmental stages (for each cultivar—closed flower: C, open flower: O, 1-mm embryo: 1, 5-mm ovule: 2 (Supplementary Fig. S1)) and leaf (L). The relative expression of *MiRWP* was calculated relative to the expression of Actin. The *Actin* gene was used as an internal control to normalize the data. Error bars representing standard deviation were calculated based on three technical replicates.

Expression profiling of *MiRWP* was performed using quantitative RT-PCR in a few monoembryonic and polyembryonic mango cultivars (Fig. 4) during flowering and early embryo development (Supplementary Fig. S1). We specifically used accessions with diverse genetic backgrounds as we believe these present the variation of the phenotype. As we defined the stages by the ovule size, the biological plan may be slightly different between the different accessions affecting the peak in expression. In polyembryonic cultivars, the expression of *MiRWP* was higher in the early stages of seed fruit development (ovule size of 1–5 mm) compared to other tested tissues and higher in comparison to monoembryonic (~6.5-fold upregulated in ‘13-1’, 4.1-fold in ‘Sabre’, and 3.8-fold upregulated in ‘Kensington Pride’ (KP)). These data suggest that *MiRWP* is acting following fertilization, as there is a correlation between *MiRWP* expression and the polyembryonic phenotype.

### Sequence variation between mono- and polyembryonic accessions in the *MiRWP* gene coding and promoter sequences

**Figure 5:**
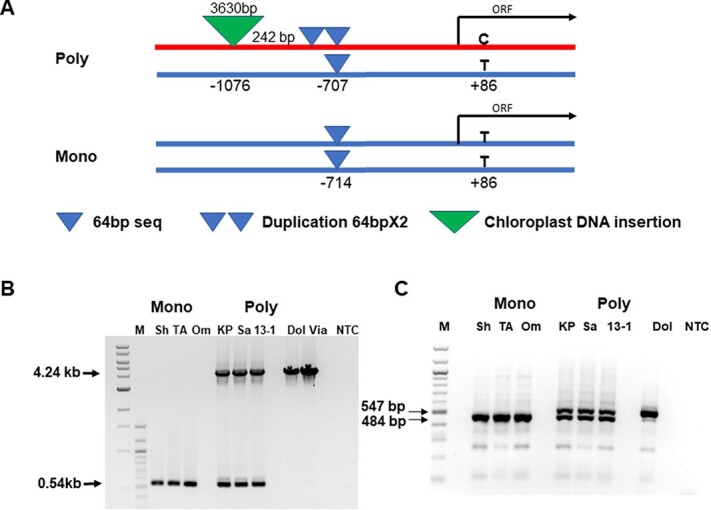
The structure of the MiRWP gene and promoter in the polyembryonic and monoembryonic accessions. (**A**) Schematic structure of gene and promoter of TA (monoembryonic) and KP (polyembryonic) accessions based on Sanger seq 1.**3** kb of promoter and the coding regions of *MiRWP* (genomic DNA). The polyembryonic allele is marked in red. The predicted ORF is marked by a black arrow. Direct duplication of 64 bases at bp −707 of the gene is indicated as two blue triangles. Insertion of a 3-kb chloroplast sequence positioned at −1076 is indicated as a green triangle. (**B**, **C**) DNA fragment PCR with several primer sets demonstrated the presence of the inserts in different polyembryony vs. monoembryony accessions (Supplementary Table S6). Samples were PCR with specific primers (RWPF9-RWPR9 64 bp duplication, RWP-F7 RWP-F8 Chloroplast insertion) and ran on 0.8% agarose gels. Monoembryonic accessions: Sh - ‘Shelly’, TA - ‘Tommy Atkins’, Om - ‘Omer’. Polyembryonic accessions: KP - ‘Kensingston Pride’, '13-1', SAB - ‘Sabre’, DOL - ‘Dolores’, VIA - ‘Vianato’. NTC - PCR negative control.

We utilized a draft of the polyembryony KP genome assembly to explore the molecular basis of the overexpression of *MiRWP* in polyembryonic accessions; TBLASTN searches [34] in the green plant subset of GenBank were used to locate scaffolds containing homologs of the polyembryony-associated citrus RWP-RK domain encoding protein *CitRWP* (GenBank accession: XP_006474671.1) and *MiRWP*. Also, 1.3 kb of the promoter of KP is presented in Supplementary Fig. S3 (TA is not shown). Comparison sequences of the coding region sequences of *MiRWP* in TA and KP identified two SNPs in the coding region in agreement with the NGS data (Supplementary Dataset S1); SNP in position 4598409, which leads to amino acid change (leucine to serine), is heterozygous in KP (polyembryony) and homozygous in TA (monoembryonic). Analysis of this SNP on the germplasm collection found that all monoembryonic varieties were homozygous, with a T/T in this position. Polyembryonic varieties had either C/T in this position or C/C. One polyembryonic accession, ‘Asam Ramuk’ (ASR), had TT at this position. Another SNP in position 4599348 that differed between polyembryonic accessions and monoembryonic accessions did not lead to a change in amino acid. SNP 4599348 is heterozygous in all polyembryonic varieties that were tested and homozygous in all monoembryonic varieties that were tested. This SNP marker and the one defined earlier at 4598161 bp could be used to screen for the polyembryony trait. Two major differences were discovered in the promoter region (1.3 kb from the ATG): KP promoter is heterozygous for a 64-bp duplication in position −714 of the gene and contains an insertion of a 3.6-kb chloroplast originated sequence at −1076 bp, relative to the start of the putative coding region (Fig. 5A and B; Supplementary Fig. S3). We utilized DNA fragment PCR analysis by amplifying the regions around the 64-bp repeat and the chloroplast insertion to validate these results in other accessions. All monoembryonic accessions tested (17) did not harbor the duplication at −714 bp or the chloroplast insertion at their promoters. Most polyembryonic (17) accessions were heterozygous for the insertions, but some were homozygous (as could be detected based on their haplotype). A few examples of the differences in the promoter region between monoembryonic and polyembryonic accessions are demonstrated by PCR of different areas in the promoter region (Fig. 5B). The homozygous polyembryonic accessions based on the SNP analysis (Supplementary Fig. S4) were also homozygous for the insertion. Only one polyembryonic accession from the ones that were tested, ASR, did not carry the 64-bp duplication and contained only the chloroplast insertion. Based on these results, it seems that the polyembryony trait is caused by the chloroplast insertion in the promoter of *MiRWP*, leading to overexpression during early embryo development.

**Figure 6:**
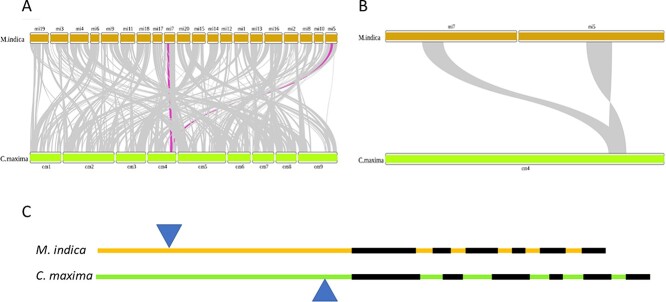
Polyembryonic trait-locus comparison between pomelo and mango genomes. (**A**) A bird-eye view of the synteny between pomelo (*Citrus maxima*) and mango (*Mangifera indica*) after the whole-genome duplication in the mango lineage (polyembryonic trait locus is highlighted in violet). (**B**) A zoom-in to the polyembryonic trait locus, where the locus in chromosome 4 of the pomelo genome was duplicated and corresponded to chromosomes 7 and 5 in the mango genome. The locus in chromosome 5 of the mango genome went through an inversion event. (**C**) The insertion in the promoter of the pomelo *CitRWP* orthologous is located in a different position and had a different sequence than the insertion in the polyembryony mango allele.

### C**haracterization of chloroplast insertions in *Mangifera* genomes**

The 3.6-kb chloroplast insert identified in the *MiRWP* promoter region of scaffold 75131_rc from the polyembryonic *Mangifera indica* variety KP was found to have the highest homology with the chloroplast genome of *Mangifera odorata* (total BLASTN score: 6274) but was not an exact match (98.43% identical). The fragment appeared to be derived from part of the coding region of a hypothetic RF1 protein (YP_010485880.1) with an unassigned function. Subsequent homology-based searches with the *M. odorata* chloroplast genome revealed that chloroplast DNA insertions were widespread throughout the chromosomes of the monoembryonic *M. indica* variety ‘Alphonso’ (Supplementary Fig. S4). In ‘Alphonso’, a maximum insert size of 2781 bp was observed with over 900 identifiable regions (e value <1 × 10^−20^) from different parts of the *M. odorata* chloroplast genome (covering 70% of the chloroplast genome).

### Independent insertion events in the regulatory regions of *CitRWP* and *MiRWP* lead to polyembryony phenotype

Following the divergence of *Citrus* and *Mangifera* (72 million years ago on average; http://timetree.org), a whole-genome duplication (WGD) occurred in the *Mangifera* genera [32, 33]. Based on our analysis, the synteny between *Citrus maxima* and *M. indica* has been conserved in 484 blocks retaining 15330 genes. The collinear blocks covered 362 Mbp of the pomelo genome corresponding to 62 Mbp of the mango genome (Fig. 6A). This might indicate further rearrangement, which broke the blocks of the mango genome after the WGD event. For example, the pseudo-chromosome 4 of the pomelo genome corresponded to a few blocks in 8, 9, 10, 14, 17, 19, and 20 chromosomes and two regions of many consecutive blocks on chromosomes 5 and 7 in the mango genome (Fig. 6B). Within the major synteny regions, two orthologous *CitRWP* genes reside, *Manin07g005330.1* and *Manin05g003710.1*. To examine whether the similarity is a result of orthology, we conducted a reciprocal best-hit analysis of BLAST using SwiftOrtho [35]. The pomelo gene *Cg4g018970*, which is the gene ID referred as *CitRWP* [19], was found to be the ortholog of *Manin07g005330* (data not shown). The collinear block on chromosome 5 went through an inversion. Based on the maximum parsimony principle, this block is more recent, probably as the WGD outcome. Within the synteny block in chromosome 7, the *MiRWP* orthologue (*Manin07g005330.1*) obtained an insertion in the promoter region (Fig. 6C). The insertion is not a miniature inverted-repeat transposable element (MITE) as in the *Citrus* orthologue and is unique in this paralogue. Therefore, we suggest that, in mango, a recent independent insertion event had occurred, which converged into the same functionality as in citrus and resulted in the polyembryony trait.

## Discussion

In this paper, we mapped the gene that causes the polyembryony trait in mango. *MiRWP* is orthologous to the *CitRWP* gene that causes polyembryony in citrus. This is one of the first examples of mapping a genetic trait in mango. The molecular and genomic tools developed for fruit trees in the last decade open a new venue for genetic analysis of fruit trees that will enable exploring the molecular basis of different traits. One of the limiting factors in fruit production is the long juvenile period from germination to fruit production - 3–10 years in mango [36] and 6–7 years in citrus [37]. Long juvenility periods slow down the breeding process and the development of large populations required for genetic analyses. The mapping of the *MiRWP* gene combined an F1 mapping population [29] and germplasm collection genetic analysis to define the associated genome region. The genetic population defines the trait’s nature, but its size did not contain enough recombinants for fine mapping. Therefore, a germplasm collection has been used to identify the causative gene. The analysis was based on 15 genomes (Supplementary Table S2) of mango that were re-sequenced, compared to the reference genome of TA, and allowed us to define a subset of SNPs for further analysis. The use of germplasm analysis can bypass some of the limitations of genetic mapping in fruit tree genetics, as large germplasm collections are readily available for many fruit trees and can compensate for the small size of the populations available. However, part of the limitation of this approach is that the genetic basis of a trait in the germplasm collection cannot be clearly defined. The mixed approach used in this paper, using both small genetic populations and an extensive germplasm collection, has some advantages as it allows us to define more clearly a probable genetic basis in a specific genetic population. Trait mapping was done by germplasm collection of diverse genotypes and linkage disequilibrium analysis.

We sequenced the KP and TA promoters to explore the molecular basis of polyembryony in mango. We defined several differences between the *MiRWP* gene and promoter region between polyembryonic and monoembryonic accessions (Fig. 5). *MiRWP* is expressed at a higher level during flower and early embryo development in polyembryonic accessions than in monoembryonic accessions (Fig. 4). As the expression experiment was based on RNA from a complete organ (flowers, fruitlets, or entire embryo sacs) but embryos develop at a much-defined region of the nucellus, we assume that the difference in expression in these specific tissues/cells is much higher. The gene is expressed in low levels in monoembryonic lines, probably only in defined cells of the ovule, and maybe specifically at the megaspore or the zygote, triggering the development of single embryos. In polyembryonic accessions, we assume that the *MiRWP* is mis-expressed also in nucellar cells, inducing additional somatic embryo development, which creates the phenotype of polyembryony (Figs. 1 and 2). As known from other development regulators such as Baby Boom or Whuschel in *Arabidopsis*, their overexpression or mis-expression can lead to the creation of embryos in different tissues [38, 39]. A similar mechanism of action was suggested for the citrus *CitRWP* polyembryony phenotype in citrus [19] and for the PAR gene in dandelion [40]. Based on sequencing of the gene and promoter of KP and TA, we performed (Supplementary Fig. S3) DNA fragment PCR analysis of the set of accessions using specific primers around the repeat and the chloroplast insertions and identified two major structural differences between the promoter region of polyembryonic and monoembryonic accessions: a duplication around −700 bp of 64 bp (which does not exist in the ASR polyembryonic accession) and insertion of 3.6 kb of chloroplast DNA around −1100 bp (Fig. 5, Supplementary Fig. S3). Within the coding region, we identified a nucleotide change at position +86 that generates a change in the protein’s amino acid. This change defines a difference between polyembryonic and monoembryonic lines that is conserved in all accessions tested except ASR. These results define two types of alleles that cause polyembryony: The major one has a chloroplast insertion, 64-bp duplication, and a change in the coding region and the minor one has only a chloroplast insertion (ASR). The ASR allele defines the importance of the chloroplast sequence insertion as the cause of the overexpression that leads to the polyembryony phenotype. As ASR is different from other *M. indica* accessions and maybe represent a different *Mangifera* species, it may represent a different allele of *MiRWP*. Further experiments to test the insertion effect or mis-expression of the gene by transgenic or gene editing analyses are required as direct proof for the causal role of the *MiRWP* promoter insertions in the polyembryony trait. However, as there are no tools for transformation in mango, and due to the long plant juvenility, an approach including complementation or disruption of the *MiRWP* gene is out of the scope of this study.

Transfer of organelle DNA fragments to the nuclear genome is frequently observed in eukaryotes [41]. We found that chloroplast DNA fragments are profusely integrated into the *M. indica* genome and describe a novel example whereby integration may act to influence sexual reproduction directly. The integrated chloroplast fragment associated with polyembryony in this study was found in both *M. indica* and *M. odorata*, thereby indicating that integration occurred before these *Mangifera* species diverged. The fragment was also found to be most homologous to the chloroplast genome of *M. odorata*, thus providing additional clues as to its ancestral origin.

The data we accumulated (Fig. 4B, Supplementary Dataset S2) demonstrate that most polyembryonic accessions are heterozygotes for the *MiRWP* allele. However, some were homozygotes for the polyembryony trait (most of them besides ASR are of Southeast Asia origin, such as Banana Long, Chokanan, Xoi Thanh, Ca Keov, and Saveoy) (Supplementary Dataset S2). The homozygous South Asian polyembryony accessions may suggest that the source of the polyembryony trait is southeast Asia, as was suggested before [20, 21]. In citrus, homozygous polyembryony was also found in low frequency [24]. One explanation could be that there is a substantial selection against it; another explanation could be that most progenies from polyembryony seeds are heterozygous and the chance to get a zygotic homozygote seed is quite rare (assuming self-pollination, only a quarter of the zygotic seeds can be polyembryony homozygous). Further phenotypic characterization and genotypic analysis is needed to understand if these homozygous accessions have a different phenotype than heterozygous ones and if there is selection against the homozygous genotype that will explain the low number of homozygotes in the germplasm. Another level of variation in the polyembryonic trait is the number of embryos per seed (from one or two to more than 10 embryos per seed). In addition, some varieties give rise to one embryo in total - but could be polyembryonic (after degeneration of the zygotic one) or zygotic. This phenomenon still needs to be further explored, as it is important for the ability to induce polyembryony and use it for agriculture or biotechnology. This probably suggests that additional factors, genetic as well as environmental ones, influence the polyembryonic trait.

Two independent insertion events occurred in the citrus and *Mangifera* lineages. In the citrus lineage, the MITE was inserted after the divergence of citrus and *Mangifera* genera; otherwise, the MITE would be found in both *CitRWP* and *MiRW*P orthologues. The insertion in the *MiRWP* promoter is of chloroplast origin which occurred after a whole-genome duplication that the mango genome went through. Otherwise, the same element had been found in the *MiRWP* paralog. The independent events converged into enhanced gene expression, which caused the polyembryony trait.

Convergent evolution may appear in different forms and usually indicates adaptation [42]. The convergence may come from different pathways that converge into the same function [43], different enzymes that may lead to the same metabolite, or different metabolites that can result in similar functions [44]. The result of this study is an example of regulatory convergence: two insertions that converged into the same regulatory effect. Further study on natural populations could be done to measure the level of adaptation.

Besides its interesting biology, sporophytic apomixis is a very valuable trait for agriculture as a method to duplicate identical copies of plants through seeds. Most crops do not possess this trait. The similar mechanism for polyembryony occurring in two different plant species, mango and citrus, suggests a common and universal mechanism that may be transferable to other species, possibly creating a new horizon for novel propagation in other plants. The dominant phenotype of the citrus and mango alleles and the ability of their *Arabidopsis* orthologue to induce somatic embryos support this idea. The challenges ahead include applications to induce embryos in other species with the mango and citrus genes or their homologs and defining the suitable tissues/promoters to test for the creation of polyembryonic seeds. This approach can open a new way to create cloned plants through seeds.

## Materials and methods

### Phenotyping of the polyembryony trait

Seeds from mature fruits (at least five fruits/accession) were collected from germplasm collections in Israel and Australia. The hard shell was carefully opened. Accessions were defined as “monoembryonic” when only a single embryo was detected. Accessions were defined as “polyembryonic” if, at least in some of the fruit, multiple embryos were detected. These tests were repeated over at least two seasons. The accession phenotypes are presented in Supplementary Table S1.

### Histological studies of embryo developmental stages

For embryological studies, young fruitlets and ovaries at various developmental stages from four mango cultivars were collected: ‘13-1’ and ‘Sabre’ (polyembryony); ‘Shelly’ and ‘Omer’ (monoembryonic). Samples of closed and open flowers, and fruitlets at sizes of 0.3 mm, 0.5 mm, and 1 cm from each poly- and monoembryonic mango cultivar (Supplementary Fig. S1) were fixed in FAA solution (4% formaldehyde, 5% glacial acetic acid, 50% ethanol) and stored at 4°C. The selected samples were passed through an ethanol series (30%, 50%, 75%, and 90% for 2 h and 100% overnight). The dehydrated samples were passed with Histoclear for clearing the tissue and finally embedded in wax. Serial sections were cut at 8-μm thickness (Leica, RM2245 Microtome) and dewaxed using Histoclear, followed by dehydration through graded alcohol series. The samples were stained with safranin for 20 min, followed by a Fast Green stain for 5 min. The sections were cleared again with Histoclear and mounted in DPX mount. The sections were examined on a Nikon Eclipse Ni-E microscope and images were taken using a DS-Ri2 camera (Nikon, Japan).

### Mango genome sequencing and variation discovery

The genomic DNA of 15 mango accessions (Supplementary Table S2) was extracted as described in Sherman et al. [28]. Whole-genome sequencing was performed using Nova Seq 6000 based on Illumina 150 bp paired-end protocols (Macrogen, Korea). On average, ~30 Gb were generated per accession. The reads were mapped onto the mango TA reference genome [33] using the Burrows–Wheeler Aligner MEM software 0.7.12-r1039, with its default parameters [45]. The resulting mapping files were processed using SAMtools/Picard tool [46] for adding read group information, sorting, marking duplicates, and indexing. Then, the local realignment process for locally realigning reads using the RealignerTargetCreator and IndelRealigner of the Genome Analysis Toolkit version gatk4-v4.1.3.0 was used [47]. Finally, the variant calling procedure was one using the HaplotypeCaller of the GATK toolkit (https://gatk.broadinstitute.org/hc/en-us). Only sites with DP (read depth) higher than 20 and MAF (minimum allele frequency) higher than 0.05 were further analyzed.

### KP genome assembly

KP DNA was extracted by BGI Genomics, Shenzhen, China. DNA libraries with 170, 200, 500, and 800 bp inserts and mate-paired libraries with 2000, 5000, 10 000, and 20 000 bp inserts were constructed for sequencing on the Illumina HiSeq4000 platform according to the manufacturer’s protocols (Illumina, CA, USA). Also, 125 bp paired-end reads were generated from the 170, 200, 500, 800, 2000, and 5000 bp libraries and 50 bp mate-paired reads were generated from the 10 000 and 20 000 bp libraries. Assembly was done using SOAPdenovo2 and SSPACE2.0. Dovetail Genomics Chicago library construction and HiRise scaffolding were used to improve the initial assembly. DNA was extracted from KP leaves using a CTAB-based method with magnetic beads [48]. A Chicago library with ~325 bp inserts was prepared from the DNA. Paired-end reads (150 bp) were sequenced by using the Illumina NovaSeq6000 platform. Then, 306.43 Gb of raw data (~635X genome coverage) was used to re-process the initial assembly using the HiRise scaffolding pipeline. Initial assembly of paired-end and mate-paired Illumina short reads with SOAPdenovo2 and SSPACE2.0 resulted in a 477.83-Mb genome with an L50 of 315 scaffolds and an N50 of 0.398 Mb. Subsequent re-assembly using a Dovetail Genomics Chicago library and HiRise scaffolding resulted in a significantly improved draft genome of 478.42 Mb in length, with an L50 of 33 scaffolds and an N50 of 4.083 Mb.

### SNP identification and genotyping of the germplasm collection

A subset of the genomic variation on chromosome 7 around the previously identified polyembryony locus (3100998–5841341 bp [30]) was extracted from the SNP analysis described above (Supplementary Dataset S1). SNPs were analyzed based on the working hypothesis that monoembryonic accessions are homozygous and polyembryonic are heterozygous or homozygous for the other allele [30]. This analysis defined two areas where most of this variation exists (between 3260000 and 3770000 bp and between 4260000 and 5200000 bp). SNPs that cover these areas were chosen based on the following criteria: (1) 30 bp, 5′ or 3′ around the SNP, there is no other SNP; (2) the 200-bp sequences (100 bp 5′ and 3′) are unique in the mango genome draft [33] based on BLASTN. SNP-type assays were designed by D3 SNP assay design (www.fluidigm.com). The SNP-type assays were used to explore the genetic variation in the germplasm collection using 96 × 96 arrays utilizing the Fluidigm EP1 device (Supplementary Table S1).

### Quantitative RT-PCR for gene expression analysis

Total RNA was isolated from various developmental stages of the flower (open and closed flower), embryo developmental stages (1-mm ovules and 5-mm ovules), and young leaf (Supplementary Fig. S1) using an RNA isolation kit (Norgen Biotek, Canada). DNA contamination was removed by treating with DNase I (Norgen) at 37°C for 15 min. First-strand cDNA was synthesized using 5X All-In-One RT Master mix (ABM, USA) by incubating at 25°C for 10 min and 42°C for 50 min, followed by inactivation of the enzyme at 85°C for 5 min. Real-time PCR amplification was carried out with gene-specific primers (Supplementary Table S3) in the ABI StepOne instrument (Applied Biosystems, USA) in three technical replicates for each biological triplicate. Expression value was normalized with endogenous control (Actin gene). The expression value for each gene (2^–ΔΔCT^) was calculated.

### Analysis of promoter region of *MiRWP*

To validate the results from the KP assembly comparison to the TA genome primers that were designed based on the TA genome draft [33] (Supplementary Table S3) on the genomic area of the *MIRWP* gene and 1.3 kb promoter (positions 4596985–4599982 TA genome), PCR and sequencing were performed on genomic DNA of two accessions, TA (monoembryonic) and KP (polyembryonic). The full sequence identified a few SNPs and two major structural differences between these two accessions. A heterozygous 64-bp duplication around −714 bp exists in KP but not in TA. Another difference is the heterozygous chloroplast DNA insertion around −1020 that exists in KP and not in TA. The sequence of the gene and promoter area of KP is presented in Supplementary Fig. S2.

### Characterization of chloroplast insertions in *M. indica* genomes

Homologous regions between various *Mangifera* scaffolds, chromosomes, and plastid genomes were identified using BLASTN [34]. Complete chloroplast genome sequences from ‘Alphonso’ (CM021858.1) and *M. odorata* (NC_066470.1) were retrieved from the GenBank database [32]. ‘Alphonso’ chromosomes 1 to 20 (GCF_011075055.1) and its associated mitochondrial genome (CM021857.1) were similarly retrieved [32].

### Synteny analysis with *Citrus*

Whole-genome proteins set of *C. maxima* and their genomic position (gff file) was downloaded from the Citrus Genome Database (http://www.citrusgenomedb.org/Citrus_maxima/C.maxima_Hzau_v1_genome/annotation/) and the set of *M. indica* together with their genomic position (gff file) from MangoBase (https://mangobase.org/easy_gdb/index.php). A BLAST was runoff one set against the other. The outcome was applied as an input to MCScanX [49]. Proteins’ genomic positions on the genome were converted to BED format from the “gff” files provided with whole-genome protein files. The output of MCScanX was read into the R environment. Synteny graphs were drawn using Rideogram [50].

The sequences of *C. maxima* proteome [19] and the sequences of *M. indica* proteome [33] were included in one file and flagged by their origin. All-against-all sequence similarity was run with BLASTP and the result was analyzed by the algorithm described in SwiftOrtho [35]. The cutoff for minimum alignment coverage was 0.5 as recommended.

## Supplementary Material

Web_Material_uhad227

## Data Availability

The data underlying this article are available in the article and its online supplementary material.
