# Supplementary material for: Promoter insertion leads to polyembryony in mango — a case of convergent evolution with citrus
Source: Hortic Res. 2023 Nov 8;10(12):uhad227. doi: 10.1093/hr/uhad227 (PMC10709545; doi:10.1093/hr/uhad227)
Supplement: Web_Material_uhad227 [file web_material_uhad227.zip › Supplementary Fig 3.docx]

Kp1

>KP1_11736 2840 bp

TCACCGCCCATATCCTTATACTAGCACCATAAACATCATCCCTAACACTACCATGAGCAC

TGTTGTAATAGCCAATATTATGAAACCCTCATCCTCAGCTACATCAACATCACCATCTCT

AATACATGTATCCCTACAATCACCATCACCAACCTTCACAGTGTCACCGTACCACTACTT

TTGTTCCAATACTATTGTAATCAACATGTTTCAACCACCACCTCCATTGTCATCAAGTCA

CCACCATTAACACTGTTGCTAACACCTATCATTAACATCCACAACAAGAAGTCTTAAGGA

AAATTGAAAAGAAAAATTATTCATAGACAATAGGGATAACACCCATATATCATATCACCA

CACTTTAATTGCGAACACCATCTTGATATTGTTGGTGTCGTCACCTTTATTATCACCACC

ACCAACAAAAATGACAATAAAATGAATTCTAGAACTTGTTGTCACGTTAGCCACCACCAC

CATAAAAAAGGATTCACAAATTGCAGCCCACCCGCTACCAATATCCAACCTTATTCCATT

ATCACTGTAGGAACACTTCAATGGGTTTGGCGGAACTTGTTAGATTTAGGATCTATCGAG

ACTAAAATTAAACAAAAATCCTTCATGAAGTTGTTGAGTTCATCGATCCTGTCTTCAATA

GAGGAATTGGAGCCATCAAAATTGGAGACACTATTATCTAGAAGAGGCTTTGATGGTGAA

GGAGATGTTTGGGTTCATCCATCTTAGCTTGTACCTATGTCGACATAAATGTAAATTTGG

TGAAAAAAGAAGCGAGATTTATAAAATAAGGTAAATTCAATAATTTAAATCTTTGTATTA

TGTATGATCATATTTATGTTAATAATATAAGTTTTAAATGGGAAAATATGATTTATATAT

TTAGTAAATAAAAAATATTTTAGGCCAAAATCCCTTTATGAGAAAATATTAATCATTCTA

TATTTATAAAAAAAACAAGAGTTAATATTTCATTATATTCACTCTTCTGTGGGGTTTATG

GGCAAATGTCCCTTATGTATCTTATCAGCTAACGATGTAGAATTTGTATTGCATGTCTAG

CTACCTTACCATGGAATTTATACCACCAAATCAGCACTCACAAACTTATTCAAAAACTTA

ACCGTTTATTTCACAACGATAATTTAACTGTCTCTCCGTCGCGCCCCTTTTAAACTCTCA

CAATTACGATCCTTACAATCATTCTTTTCATTCGCACCAGTGGCGGTGCTTCTTCATTGC

GCCGCCACCATAACCAAATATCATCATCAGCATCATCATCCATAAATCTTCACTCTGAAA

TTTATATAACATCTCATGCATGGCAGATTCCAAGTCCAATGTCTCCTACCAGGATCCACA

TGATGCCTCTTTCATCGATGACGTTGTCAATTACATCAACAATTTAACTCCTTGTGTTGA

TGATTTATCACTTTTTGAGGACCCTTCTCTGCTTCCATCTACAAGTAATCCTGTTAACAT

TCAAGATGCTGATGATTCCTTTAATGATCCAGTTTTCTGGGATATTGTTAATAACTTCAA

TGGAGGGAGTTCTCAAGTGGGTCCATTGGAACATCGCGTTGAAACGAGTCATCAGGAGTG

GCTTGAGAATGATACTGGTGGGAATATGAGGACAATGCCAATGTGGCCAGTTCCGGCCGT

GCCATATTTATGTAGTTGTTGCCTGGTGCTTAGAGAAATAATTCATACAAATGGTATATG

TTTTTGTTACTTTAAAGGGTAGTATTTAAAGTGAAATTAAACACTTTTTGTTTAGTTTAA

TTTGGTGCTGATGGTGTAGGGTTTGAAACTACAAAACTTGAAATTCATGGAAGACTTGGA

ATGATCTGTCATGCTATTCTTGAGATTCATCAAAGTTTTGCTGTTTTTGAGTATCAAATG

ATTGAGTAAGTTTCTATGTGAGTCTGACTATATTTCATCTCTTGTAGTTCTTATGATTTT

GACTTTGAAGTTTGAACCGCTCTTCTTCTCTTTCATCAGTTTCTGCAAGAAAAGCATAGA

GGATGTGAAGAATTTTCTGGTTCAATACTGTGATGAGCGGAAACATGCAGGCTTCGTCAT

GACAAGGGATCCCTTATCAATCTTCTATGAAGCCGTATGTGTTGGCTTGGACAGGGAAGA

AAATTTAACAGCAGAGGACTTGCCTCAACCTCCACCTCCACCCACCACCTCAGGTGCCTT

TGTGTTTCTTTACCTTCAAAAGTTCTTTAGATTTCATGTTATATATAGAATATTTCTCAA

TGAATGTTAAAATATGTTTAGTTGCAAATGAAATGAATCAGCCAGAGTCAGAGAAGGATG

GGGAGAGGCCTCCGAGGACTTCCCTTGCGTTACAGGTTACATAGTCTTTCTCATATCTTC

AATTTTTTGCCATCTGCATTGATTTTCTAACTAGGGAAATTGTTTTTGTGGGAGGGATTT

GTGCAGAGGGAGAGAACAGGGAAATTGACATTGGGAGACTTAAGAAATTATTTTCATCTT

CCCATTGAGGAGGCGGCTAAGAGATTGAGATTATGTCCCACTGTAGTGAAGAAGATATGC

CGCAGATATGGCTTACTCAGATGGCCTCACAGAAAGGTTTCTGTGTTCTACTCCCTTCTT

CTTCCTTCTAATCTGATTAACATCATATCTAAAATGTTGTGTCATGCTGGTATAAGCAGA

TCAAAAGCCTGGAGAGGCAGATACAAATTGCAACCCCGAGATTAAACTCAATTGATCCAG

CAGAGAGGGCAACTGCTGAAGCTGAAATCCAGAGGCTTCGACATGAAATTGATATTGCTT

GTACTGGCAGAAAATCATGA

KP2

>seq_12287 6512 bp

ACATCATCCCTAACACTGCCATGAGCACTGTTGTAACAGCCACTATTATGAAACCCTCAT

CCTCAGCAACATCAACATCACCATCTCTAATACATGTATCCCTACGATCACCATCACCAA

CCTTCATAGTGTCACCACCACCACCACTTTTGTTCCAATACTATTGTAATCAACATGTTT

CAACCACCACCTCCATTTTCATCAAGTCACTACCATCAACACTGTTGCTAACACCTATCA

TTAACATCCACAACAAGAAGTCTTAAGGAAAATTAAAAAGAAAAGTTATTCATACGTCAG

ATCACCTTTATTCGGCAAGAGCGAAATCCTTCTCAGTCATCAAAATATCCAGACGCATTT

CTTCCCTTTGAATATAGGATATAGCAATTTCTCTTGGATTTATCAGTCGTAGCAGGAGAC

AACCGATTTTGATATTATTCATTACCTTTTCATTTAAAGAATTATCCCATCTCAACTGAA

AATGAAAATCTTTTTTTAGTAAGAAATCAAGCTCTGCTTCTATATTGCTCTTGTATTGCT

TTTTCTTTCTACGTTTTTTCATGTCAGATCCCGCATACTTTTCTTCAACATCTTTTTCTT

GCTTTGAGAGAACTGATCCAAGACTTACTTGGTTTTGTGCATCTGATCTCGGATTTCCTT

GGCTTGTGGTTTTTTTTTCTTCTTGACTTCCATTGTCTAATTCAAGATATTTTTTTTTAT

TCGATGATATAAAAAGATATTCCTTTTTCTTTATAGTTCTCTTTTTATTACCATTTGCAT

TTCCATTAAAATCGGAAAGAAGTAATTTGATTGGTATGATCCATGGTTTCGTTTTATATG

CATTAAAAAGTAGCACAAATTCTGGGAAGAACCAAAGCTCCCAATTTGATATAAGACTAT

TTAGTATTTTGTTGTTCATTCCCATCCAATCAAAAACGAACTCTTTCTGATTGGATGGGT

TAATTTCTTCATTTTGATGAATTGTAAAATAAAAAAGACCTTTCTTAGTAATTTTATCAA

TTATTTGATAATTATCCGCCCAATCTTAGTATTTTGATTACTGTTGGTACCAGTATCCAT

ATCAACCCAGGATTGGATATCGATTTTATTTCTAAGACAAAAATAGAGAATTCTCCAATC

AAAATATTTTCTATTCAAAATTTTATCTATATCCATAATATCGTCTTCCCCTAGATAATT

ATTGATAGGGATACTTCCCAGCATACCAAAAAATTTTCCTTTCCCTATGTTGTAATTATA

AAAACTTTCTTGGACTAGTGATCTATCAATAGACGAGTCTTTCTTATCGTCATAATTAAT

AGATTTATATGATAAAAGATCATATCTATAGTATTTTTGAAAATTGGATTTTTGATTCTG

TAATGAGTCCGCTTCAAAAAGATTTTGTTTTTCTTTTTGGTAATGAGTTAATCCGTTTTT

TTCATACGAATTTTTTTGTTTAAATCTTTATTTTGAGTCATACAGTCTTGATTCGCTCGA

TTTCGCCATTTTTGTGGTACTAATTTAGTCCATCTAAGCCTAGGTAAATCATATTGATAA

TGACTACTTAACCAGGTTTTCCATTCATTCATTCCAAACTTCCAAAAAGGTTTATGTCTT

AATTTGAAATATTCCTTTTTTTTCAAAACAATCTTTTAGTTCATTCTTAAGAAAAAGAGA

TGTTATTGAAGGACAGATCTTAAATTAGAAAAGTTAATAACTTGGGTTTGTGATAATTTG

TAAAATACATATGCTTGTGATTGTGAGAAAGAAGATAAATCCCAAAAAATCTTTGAATTC

TTATTATTATTACTAATCTTATAAAGTGATTTTTTTAGAGTCGAAAGAAAGTGAATTGTA

TTTTTAGTTGGTTTATTAATTTTTTCCTGATTTGCTTCATTATTGTGGACATTTTGAATT

TGAATTTTTTTTGTTGATTCAAAAAAAATTTTTGCATTCATTCTTGGAATATTAAGTATA

GATAAAAAAAAGTTTATGTATACCCTTTTAATCAAAAATTTTATAAAAAAATATGATTTA

CGGATTAATCGAGTATTTCTTCTTTTTAATATCTGCCAAATCTTTTTTGATGATTCTAAT

ATTTTAACACGATAACTTATTTTGGTAGAACTAATATTTATTTCTTGAGTTAGCAATTCT

TTTTTCTTCTCTTTTGTAATTTTTTCTATTTGGTTTCTAATTATGTTTGTTCTATTACTT

AGATCTTTCATTTGTTTTTCTGTTAGTGAGAAATTTGTCCAATGCATAGATCGAATTTGA

ATAGATAATTTGTTAATCGTAAGATTCCCGATTATTGTTATCGAATCTTTTTTAATTTCA

CCCAAGTCCTCTATATCTCTCGTTTCTCCCAATATAACTAATTGAATTGGCTTTCTTTTT

GAAAGTCTTTTTATTCTTCCTTTTAGAAATAGAATGCTTTTGATGACCCATTTGTTTGTT

TCTTTTGCCACTTTTCGGAAAATTTTTGTTCTTTCTTTTAAAATTCTTAAAACTATAAAA

GACTTGGTTTTCCATTTTCTAATTTTTTTTTTTGAATTCTTGCAAAACGGGTTCAAAAAA

TGAACGTTGTTTTCGGGGAGAACCAAAAGGAAATTCAGTTTCCATTCCCAAAACTGTTAA

AAAACAAAAATCACTTTCTTGTCCTTTTATTTTTTTCAATGAATCCTTATGTAGGGATTG

TAGCTTAGGTCTGCGCCGGGATTGTAGGGAAAAAGGAAATAGGATCTTTATTTGAATACC

GCCTGTTAACCAGTTATACGGAAATTCTGTTTCGGATAATTGAATCCCATTATAGGTGCA

TTTAACATGCATTTCCTTTTTCCAATTATTTAAATCCTGGGACCACCCGGGCCATTCAAA

TAATAGTATGCGAGCGTGATTTTTAGCTATTATCAATTAAGGTAATATAATATATTTTTT

AAGAATCGATTGAGTTAATAATAGAAACCCTCTTATTACTTGAGAAAGGAAAAAGCTATC

CCAGGTTTCTGCTATTTTCATCCGTCCTTTTTCTTTTCTTTTGTATTCTTCTTTTTTATC

AATTTTTTTTTTTCATTTCATTTGTATAATCAGACTGTTTTTGGTCTTTTTGTTTCCACA

TCCAATTTCTAAAAATTCGAAAAATTCCGTTGATCGGTCCGGAAATCTCAAAAGAAAAAT

AAAAGAGTTTCTTTATTCTGTCCAAAAAAAGGGGTGAATGGGTATTTGCTTGAAAAAATT

CCCAAGTAACGGTTTTGCGTCTTTGTGCGCGCATGGAGCCTTTGATTAGCTCTCGACGAA

AATCCGATTTGTACGAATAGCGTCTCAACTTTACTTCCTTTTTTTGCTCAAGATTCTTAT

TATATTTGTTATTAGTATAAGTAAAAATCAATATCCGTTTGACGTTTCTTGTACTAATTA

CAGGACCGTCCGCCACGTTTTCTTCGGTTTCGCTTTCCTTTTTTTTTAAATCAGACACTG

ATTTGTATGACCATCGAGGAACTTTTTTACTAATTTCTTTTATTCTAATAGATTTTTTTC

TAATTGTTTGAGCGTTGGGAATCGTTAGAACTGCATCAAATAAAAATTTGAAAATTTTGA

TTCGATATTCTGAATCAATTTTCTCTCGTTTGGGTTATGGAAATAAAGAACATACTTTTC

TTTTTTCTCTTGAAAATAATTTTCTACTCAATCTGTCTATTGTCTGTTCAAATTCGTGGT

AATCATTAATAAGAAGAAGAAAACCGTGAATCTTATTTATCCAAATGATTTCTCTGTTAT

TTTGGATATAAGTTTCATTTAGGATTACAGGTGAAAATAATTTTTTCATTCTTCCCCCAG

AAGATCCACATGTGAAAGGATCATATATTTTAGGTAAATATTCTTTTTTAGTTTCATCAT

TACATAATTTAGTCTTTTTGCTAACACCTATCATAGACAATAAGGATAATACCCATACAT

CAGATCACCATACTTTAATTGCGAACACCATCTTGATATTGTTGGTGTCGTCACCTTTAT

TATCACCACTACCAACAAGAATGACAATAAAATGAATTCTAGAACTTCTTGTCCTGTTAG

CCACCACCACCATCAAAAAGGATTCACAAATTGCAGCCCACCTGCTACCAATATCCAACC

TTATTCCATTATCATTGTAGGAACACCTCAATGGGTTTGGCCGAACTTGTTAAATCTAGG

ATCTATCTAGACTAAAATTAAACAAAAATTCTTCATGGGTTTGGCCGAACTTGTTAAATC

TAGGATCTATCTAGACTAAAATTAAACAAAAATTCTTCATGAAGTTGCCGAGTTCATCGA

TCTTGTCTTCTATAGAGGAATTGGAACCATCAAAATTGGAGACACTATTATCTAGAAGAG

GCTTTGATGGTGAAAGAGATGTTTGGGTTCATTGATCTTAGCTTGTACCTATATCGACAT

AGATGTAAATTTGGTGAAAAAAGAAACGGGATGTATATAATAAGGTAAATTCAATAATTT

AAATCTTTGTATTATGTATGATCATATTTATGTTAATAATATAAGTTTTAAATGGGAAAA

TATGATTTATATATTTAGTAAATAAAAAATATTTTAAGCCAGAATCCTTGTATGAGAAAA

TATTAATCATTCTATATTTATAAAAAAAACAAGAGTTAATATTTCATTATATTCTCCCAT

CTTTGGGGTTGATGGGCAAATGTCCCTTATATATCTTATCAGCTAATGATGTAGAATTTG

TATTGCATGTCTAGCTACCTTACCATGAAATTTATACCACCAAATCAGCACTCACAAACT

TATTCAAAAACTTAACCGTTTATTTCACAACGATAATTTAACTGTCTCTCACTCGCGCCC

CTTTTAAACTCTCACAATTACTATCCTTAGAATCACTCTATTCATTCCCACCAGTGGCGG

TGCTTCTTCATTGCGCCGCCACCATAACCAAATATCATCATCATCATCCATAAATCTTCA

CTCTGAAATTTATATAACATCTCATGCATGGCAGATTCCAAGTCCAATGTCTCCTACCAG

GATCCACATGATGCCTCTTTCATCGATGACGTTGTCAATTACATCAACAATTCAACTCCT

TGTGTTGATGATTTATCACTTTTTGAGGACCCTTCTCTGCTTCCATCTACAAGTAATCCT

GTTAACATTCAAGATGCTGATGATTCCTTTAATGATCCAGTTTTCTGGGATATTGCTAAT

AACTTCAATGGAGGGAGTTCTCAAGTGGGTCCATTGGAACATCGCGTTGAAACGAGTCAT

CAGGAGTGGCTTGAGAATGATACTGGTGGGAATATGAGGACAATGCCAATGTGGCCAGTT

CCGGCCGTGCCATATTTATGTAGTTGTTGCCTGGTGCTTAGAGAAATAATTCATACAAAT

GGTATATGTTTTTGTTACTTTAAAGGGTAGTATTTAAAGTGAAATTAAACACTTTTTGTT

TAGTTTAATTTGGTGCTGATGGTGTAGGGTTTGAAACTACAAAACTTGAAATTCATGGAA

GACTTGGAATGATCTGTCATGCTATTCTTGAGATTCATCAAAGTTTTGCTGTTTTTGAGT

ATCAAATGATTGAGTAAGTTTCTATGTGAGTCTGACTATATTTCATCTCTTGTAGTTCTT

ATGATTTTGACTTTGAAGTTTGAACCGCTCTTCTTCTCTTTCATCAGTTTCTGCAAGAAA

AGCATAGAGGATGTGAAGAATTTTCTGGTTCAATACTGTGATGAGCGGAAACATGCAGGC

TTCGTCATGACAAGGGATCCCTTATCAATCTTCTATGAAGCCGTATGTGTTGGCTTGGAC

AGGGAAGAAAATTTAACAGCAGAGGACTTGCCTCAACCTCCACCTCCACCCACCACCTCA

GGTGCCTTTGTGTTTCTTTTCCTTCAAAAGTTCTTTAGATTTCATGTTATATATTGAATA

TTTCTGAATGAATGTTAAAATATGTTTAGTTGCAAATGAAATGAATCAGCCAGAGTCAGA

GAAGGATGGGGAGAGGCCTCCGAGGACTTCCCTCGCGTTACAGGTTACATAGTCTTTCTC

ATATCTTCAATTTTTTGCCATCTGCATTGATTTTCTAACTAGGGAAATTGTTTTTGTGGG

AGGGATTTGTGCAGAGGGAGAGAACAGGGAAATTGACATTGGGAGACTTAAGAAATTATT

TTCATCTTCCCATTGAGGAGGCGGCTAAGAGATTGAGATTATGTCCCACTGTAGTGAAGA

AGATATGCCGCAGATATGGCTTACTCAGATGGCCTCACAGAAAGGTTTCTGTGTTCTACT

CCCTTCTTCTTCCTTCTAATCTGATTTACATCATATCTAAAATGTTCTCTCATGCTGGTA

TAAGCAGATCAAAAGCCTGGAGAGGCAGATACAAATTGCAACCCCGAGATTAAACTCAAT

TGATCCAGCAGAGAGGGCAACTGCTGAAGCTGAAATCCAGAGGCTTCGACATGAAATTGA

TATTGCTTGTACTGGAAGAAAATCATGA
