# Supplementary figures and images for: Promoter insertion leads to polyembryony in mango — a case of convergent evolution with citrus
Source: Hortic Res. 2023 Nov 8;10(12):uhad227. doi: 10.1093/hr/uhad227 (PMC10709545; doi:10.1093/hr/uhad227)

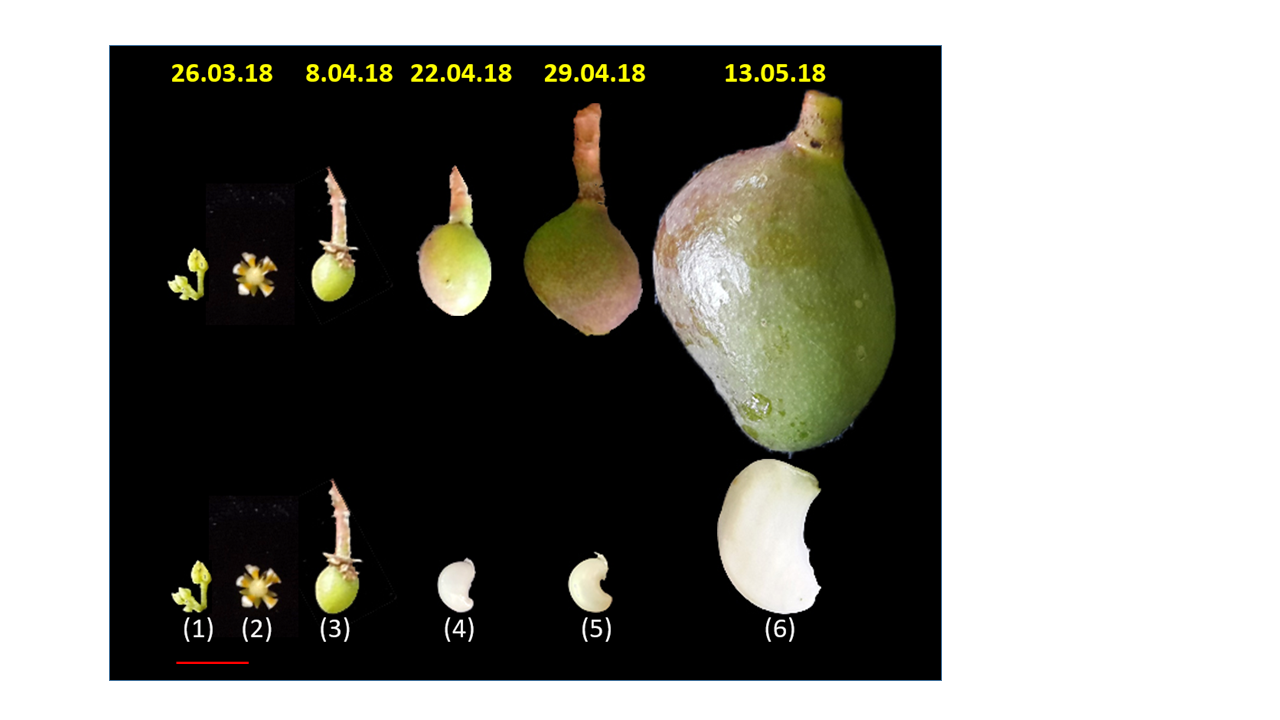

Supplement: Web_Material_uhad227 [file web_material_uhad227.zip › Supplementary Fig 1.tif]

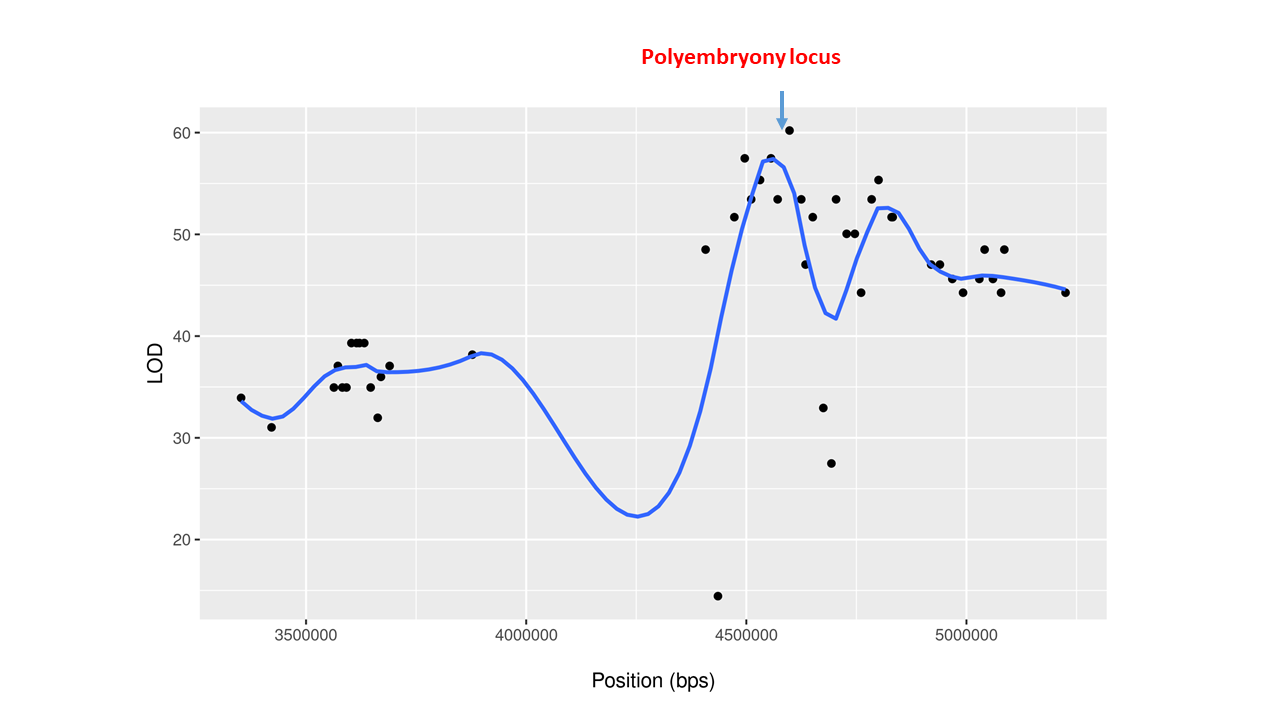

Supplement: Web_Material_uhad227 [file web_material_uhad227.zip › Supplementary Fig 2.tif]

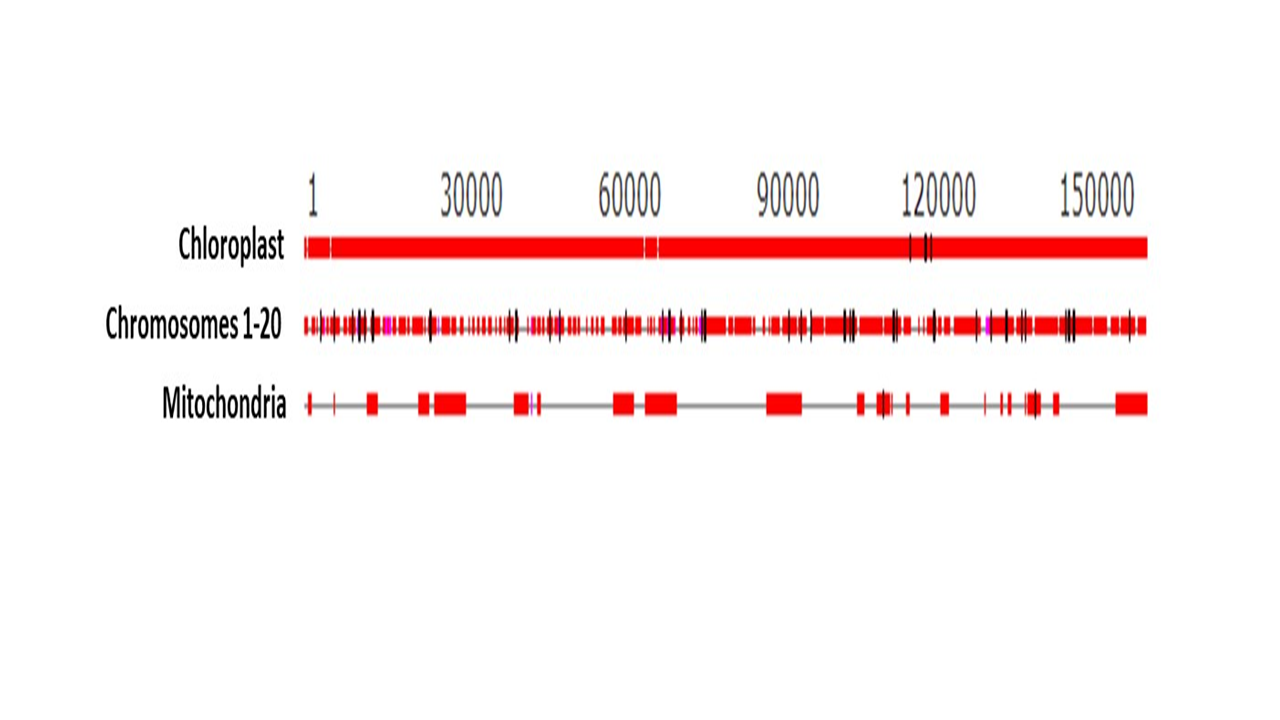

Supplement: Web_Material_uhad227 [file web_material_uhad227.zip › Supplementary Fig 4.tif]
